# Prognostic Impact of Stimulator of Interferon Genes Expression in Triple Negative Breast Cancer

**DOI:** 10.1002/cam4.70666

**Published:** 2025-02-18

**Authors:** Tetsuyo Maeda, Makiko Ono, Tomo Osako, Tomohiro Chiba, Satoko Baba, Asumi Iesato, Yukinori Ozaki, Yuka Inoue, Natsue Uehiro, Yoko Takahashi, Takayuki Kobayashi, Takahiro Kogawa, Tomohiko Ohta, Shigehisa Kitano, Takayuki Ueno, Shinji Ohno

**Affiliations:** ^1^ Breast Oncology Center Cancer Institute Hospital, Japanese Foundation for Cancer Research Tokyo Japan; ^2^ Department of Medical Oncology Cancer Institute Hospital, Japanese Foundation for Cancer Research Tokyo Japan; ^3^ Department of Pathology Cancer Institute Hospital, Japanese Foundation for Cancer Research Tokyo Japan; ^4^ Department of Advanced Medical Development Cancer Institute Hospital, Japanese Foundation for Cancer Research Tokyo Japan; ^5^ Division of Breast and Endocrine Surgery St Marianna University School of Medicine Kawasaki Japan

**Keywords:** changes in STING expression, NAC, prognosis, STING, TNBC

## Abstract

**Background:**

Patients with triple negative breast cancer (TNBC) who have a poor response to neoadjuvant chemotherapy (NAC) have worse survival and new treatment strategies need to be developed. TNBC is considered a subtype in which the cyclic GMP‐AMP synthase (cGAS) is linked to the stimulator of interferon genes (STING) pathway, an innate immune response that recognizes cytosolic nucleic acid components, is activated when DNA damage occurs, and is attracting attention as a new therapeutic target.

**Methods:**

Patients with TNBC who underwent surgery following NAC and for whom pre‐ and post‐treatment tissue specimens were available were enrolled in this study. To examine the association of STING expression with immune profiles and prognosis, STING, cGAS, CD8, and programmed cell death ligand 1 (PD‐L1) expressions in tumor cells (TCs) and immune cells (ICs), and tumor infiltrating lymphocytes (TILs) were assessed using immunohistochemistry of specimens obtained at pre‐treatment and at surgery.

**Results:**

Ninety‐one cases were eligible, of which 68 cases were evaluable and included in the analysis. The high STING expression at baseline was marginally correlated with TILs, but not with CD8^+^ cells or PD‐L1 expression. Patients with sustained high expression of STING before and after NAC had a significantly poorer prognosis than that of others for distant recurrence‐free survival and breast cancer‐specific survival independent of nodal status, lymphatic invasion and therapeutic effects (*p* = 0.024 and 0.014, respectively).

**Conclusion:**

TNBCs with sustained high STING expression following NAC demonstrated a poor prognosis and will be a target for new treatment strategies.

## Graphical abstract

Triple negative breast cancer with sustained high STING expression following NAC demonstrated a poorer disease‐free survival and will be a target for new treatment strategies.

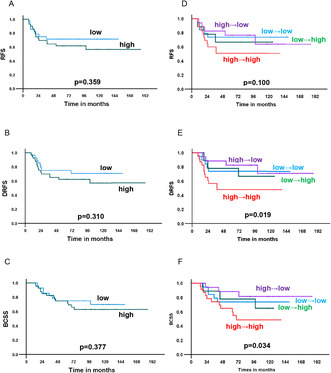

**Abbreviations**
A: anthracycline
BCSS: breast cancer‐specific survival
cGAMP: cyclic GMP‐AMP
cGAS: cyclic GMP‐AMP synthase
CR: complete response
DRFS: distant recurrence‐free survival
DSB: double‐strand break
EDTA: ethylenediaminetetraacetic acid
ER: estrogen receptor
HER2: human epidermal receptor 2
HR: Hazard ratio
HRD: homologous recombination deficiency
IC: immune cell
ICI: immune checkpoint inhibitor
IHC: immunohistochemistry
LVI: lymphovascular invasion
NAC: neoadjuvant chemotherapy
pCR: pathological complete response
PD: progressive disease
PD‐L1: programmed cell death ligand 1
PgR: progesterone receptor
PR: partial response
RFS: recurrence‐free survival
RT: room temperature
SD: stable disease
STING: the stimulator of interferon genes
T: taxane
TC: tumor cell
TILs: tumor infiltrating lymphocytes
TNBC: triple negative breast cancer

## 1 Introduction

Triple negative breast cancer (TNBC) is a highly aggressive disease that constitutes 10%–20% of breast cancer cases [1, 2]. According to age‐adjusted rate of breast cancer cases per 100,000 women SEER 222017‐2021, TNBC accounts for 13.6% of breast cancer [3]. Conventional chemotherapy is the standard‐of‐care for perioperative systemic therapy. In addition, based on the results of the KEYNOTE‐522 trial, regimens combining conventional chemotherapy with immune checkpoint inhibitors (ICI) are commonly used as perioperative chemotherapy for high‐risk early‐stage TNBC [4, 5]. While cases that achieve pathological complete response (pCR) to neoadjuvant chemotherapy (NAC) have a relatively good prognosis, cases with non‐pCR have more distant recurrence, which remains essentially incurable [2]. New treatment strategies are needed for these drug‐resistant TNBCs.

Gene expression profiling has shown that TNBC is a heterogeneous disease consisting of six distinct molecular subtypes [6]. Currently, a more precise classification into four subtypes has been proposed [7]. The majority of TNBCs are of the basal‐like type, with the basal‐like 1 type in particular being found to express genes involved in the cell cycle, components and pathways of cell division, and DNA damage response pathways [6]. DNA is constantly damaged by external and internal factors, such as radiation, chemotherapy, and DNA replication, and genome homeostasis is maintained by repairing the damages or inducing cell death when they cannot be repaired. One of the most toxic forms of DNA damage is a double‐strand break (DSB). Mutations or methylation of genes involved in the DNA homologous recombination repair mechanism results in homologous recombination deficiency (HRD) [8, 9, 10]. HRD is reportedly present in approximately 60% of TNBCs, and TNBCs show higher genomic instability than that of other subtypes [10, 11].

Stimulator of interferon genes (STING) is a protein that plays an important role in the innate immune response that recognizes nucleic acid components, and it is involved in the control of infection, inflammatory diseases, and cancer by eliciting a type I interferon (IFN) inflammatory signaling response. STING is expressed at a constant level in a variety of cells, albeit with variable expression, but its expression in cancer cells has been reported to be low [12, 13].

In cancer cells, when a DSB occurs due to chromosome segregation errors, radiotherapy, or chemotherapy, micronuclei form outside the nucleus. As DNA accumulates in the cytoplasm, the cyclic GMP‐AMP synthase (cGAS) recognizes fragmented DNA and synthesizes cyclic GMP‐AMP (cGAMP). This activates STING localized to the endoplasmic reticulum, which ultimately induces type I IFN production and increases the inflammatory cytokine expression. These cytokines recruit T cells, which trigger an endogenous antitumor immune response in which natural killer cells and CD8‐positive T cells attack the tumor cells [14]. In addition, cGAMPs produced by cancer cells can migrate to neighboring cells and antigen‐presenting cells by forming gap junctions [15, 16, 17]. Previous reports have shown that the STING pathway is activated in both the tumor and surrounding normal cells, contributing to the control of tumor growth [18, 19, 20, 21, 22].

However, some studies have reached conflicting conclusions. When DSBs occur in cancer, cGAS can be relocated to the nucleus wherein it obstructs the formation of the poly ADP‐ribose polymerase 1‐Timeless complex, and maintains chromosomal instability, which potentiates tumor evolution [23, 24]. In mice models, it has been demonstrated that STING contributes to cancer development when it causes chronic inflammatory stimuli [25]. Thus, no clear conclusion has been reached on the role of STING in cancer development and progression. Furthermore, studies to date have mainly used cell lines or mice, and there are few reports in which human breast cancer tissues were used to examine STING expression and therapeutic efficacy and prognosis.

Therefore, we used archived tissues from patients with TNBC, which have frequent HRD [10, 11] and presumably a more activated cGAS‐STING pathway, to examine STING and cGAS expression and their changes by chemotherapy in association with immune status, chemotherapy response, and survival.

## 2 Materials and Methods

### 2.1 Clinical Material

This is a retrospective analysis of tissues from a single center. Data of patients with TNBC who received NAC followed by surgery at our Hospital from May 2005 to December 2015 were extracted from the institutional database. Patients with the following criteria were excluded: had received a regimen other than anthracycline (A) or taxane (T), whose tumor volume was low and not enough for the study because of a good pathological response including pCR and residual cancer burden (RCB) 1, whose biopsy specimens were not available, had a history of other cancers diagnosed within 5 years, and who had bilateral breast cancer.

This study was approved by the institutional review board (registration number: 2018‐GA‐1220). This retrospective study did not require informed consent, and patients were given the option to opt out.

The chemotherapy regimens included four cycles of FEC (fluorouracil: 500 mg/m^2^, epirubicin: 100 mg/m^2^, cyclophosphamide: 500 mg/m^2^) or six cycles of CAF (cyclophosphamid: 500 mg/m^2^, adriamycin: 60 mg/m^2^, fluorouracil:500 mg/m^2^) alone or followed by four cycles of docetaxel (75 mg/m^2^) or twelve cycles of paclitaxel (80 mg/m^2^). The patients with stable disease (SD) and progressive disease (PD) who received only A or T as NAC were given additional T or A as adjuvant therapy. The clinical therapeutic effect of chemotherapy was evaluated using the Response Evaluation Criteria in Solid Tumors v1.1 [26], and the histological therapeutic effect was evaluated according to the Japanese guidelines for pathological assessment of therapeutic effects [27].

### 2.2 Immunohistochemical Analysis

Staining for cGAS (MB21 D1, Sigma), STING (TMEM173, Proteintech), estrogen receptor (ER; SP‐1, Roche), progesterone receptor (PgR; 1E2, Roche), human epidermal receptor 2 (HER2; 4B5, Roche), Ki67 (MIB‐1, DAKO), CD8 (C8/144B, Nichirei Biosciences), and programmed death ligand 1 (PD‐L1; SP142, Abcam) was performed immunohistochemically using biopsy and surgical specimens. The tubal fimbria was used as a positive control for cGAS and STING, and the thyroid gland as a negative control. All immunohistochemical studies were performed using formalin‐fixed, paraffin‐embedded specimens, and cut consecutively at 4 μm thick slices.

Immunohistochemistry (IHC) for cGAS, STING was performed on the Nichirei Bioscience Histostainer 36A automated staining platform. Slides were pre‐treated in ethylenediaminetetraacetic acid (EDTA)‐based buffer solution (pH of 9.0) at 95°C for 30 min. Primary antibodies (Table S1) were incubated for 30 min at room temperature (RT), followed by Histofine Simple Stain MAX‐PO (MULTI) secondary antibody (Nichirei Bioscience) for 30 min at RT [26]. IHC of ER, PgR, HER2, Ki67, CD8, and PD‐L1 was performed on the Leica BONDIII in accordance with the standard operation process (Table S1).

The scores of cGAS and STING were scaled by H‐score because the score is considered to reflect protein expression in tumor tissues in more detail by taking account of not only the proportion but also the intensity of protein expression [28, 29] The score is obtained by the formula: 3 × percentage of strongly staining nuclei +2 × percentage of moderately staining nuclei + percentage of weakly staining nuclei, giving a range of 0–300. Two trained pathology specialists read and scored STING expression in each tissue. When the scoring was different between the two, they discussed the staining and decided the score. The cut off value was selected based on the optimal cut‐off for prognostic stratification of breast cancer‐specific survival (BCSS). That is, the cut‐off value was the point at which the hazard ratio (HR) was highest when comparing between the groups. When the factor showed no correlation with prognosis, the median value was selected as the cutoff value.

TNBC is defined as having less than 1% ER and PgR and with a negative HER2 according to the ASCO/CAP Clinical Practice Guidelines [30]. The Ki67 labeling index was calculated by the global method according to the International Ki67 in Breast Cancer Working Group [31]. TILs and CD8‐positive cells were assessed according to the methods recommended by the International TILs Working Group [32, 33, 34, 35]. CD8‐positive tissues were defined as those with a value greater than 100 positive cells/field (×400) on average. PD‐L1 was assessed separately for tumor cells (TC) and immune cells (IC), and more than 1% was considered positive [36, 37].

### 2.3 Statistical Analysis

The variables were compared using two‐sided Pearson *χ* ^2^ test or Fisher's exact test.

Survival was calculated from the start of chemotherapy to the event or last follow‐up and plotted as Kaplan–Meier curves with a log rank test. HR, 95% confidence intervals, and corresponding *p* values between categorical scores were calculated using Cox regression analysis. Data were considered significant when *p* < 0.05. IBM SPSS statistics 27.0 for Windows (IBM Corp., Armonk, NY) was used to perform statistical analyses.

## 3 Results

### 3.1 STING And cGAS Expression in TNBCs

**FIGURE 1:**
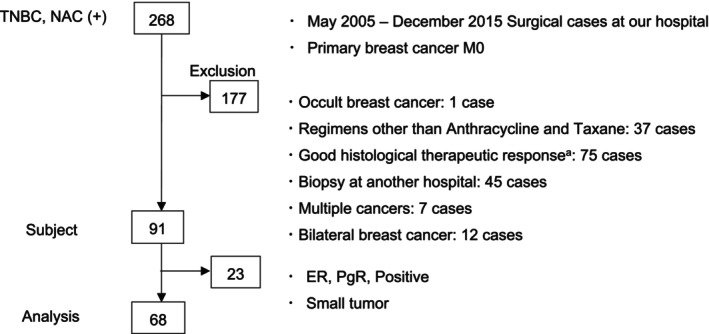
Flowchart of the study. NAC, neoadjuvant chemotherapy; TNBC, triple negative breast cancer. ^a^Good histological therapeutic response: Little or no residual tumor.

We identified 268 patients with TNBC who received NAC from May 2005 to December 2015. Of these, 91 patients were eligible for the study according to the study criteria. All eligible cases were re‐evaluated for ER, PgR and HER2 expression, and 23 cases showed a positive staining of either ER or PgR or their tumors were too small for re‐evaluation, which was equivalent to RCB 1 [38], leaving 68 cases for the following analysis (Figure 1).

**FIGURE 2:**
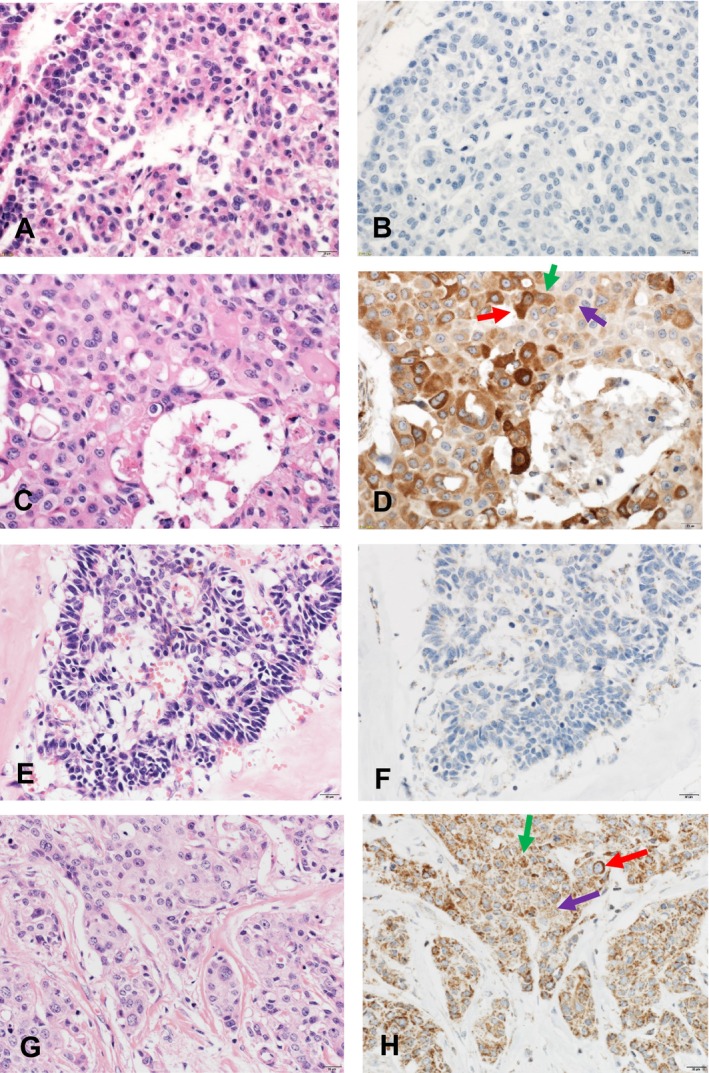
STING and cGAS expression by IHC. (A) HE (×400) staining with a representative field of cancer. (A) Immunostaining of STING in a serial section of A. Score 0: no staining in the cytoplasm. (C) HE (×400) staining with another representative field of cancer. (D) Immunostaining of STING in a serial section of C. Score 1, weak staining (violet arrow); Score 2, moderate staining (green arrow); Score 3, strong staining (red arrow). (E) HE (×400) staining with another representative field of cancer. (F) Immunostaining of cGAS in a serial section of E. Score 0: no staining in the cytoplasm. (G) HE (×400) staining with another representative field of cancer. (H) Immunostaining of cGAS in a serial section of G. Score 1, weak staining (violet arrow); Score 2, moderate staining (green arrow); Score 3, strong staining (red arrow).

Among those 68 cases, 40 (58.8%) had a high expression level of STING in TCs and 31 (45.6%) had a high expression level of cGAS in TCs before chemotherapy (Figure 2). In the adjacent tissues surrounding the cancer cells, STING and cGAS expression was almost universally high.

### 3.2 Association of STING and cGAS Expression With Clinicopathological and Immunological Factors

We examined whether STING and cGAS expression in TCs was associated with clinicopathological and immunological factors at baseline. High STING expression in TCs showed a trend toward more TILs (*p* = 0.057). STING high cases were more common in invasive ductal carcinoma (*p* = 0.049). No other associations of STING expression were observed (Table 1). High cGAS expression was more common in high clinical T stage than in low clinical T stage (*p* = 0.019) (Table S2).

**TABLE 1:** Association between STING expression in biopsy specimens and clinicopathological characteristics.

Characteristics	Total (*n* = 68)	STING low (*n* = 28)		STING high (*n* = 40)		*p*
Menopause						0.907
Pre	31	13	(41.9)	18	(58.1)	
Post	37	15	(40.5)	22	(59.5)	
cT						0.923
1c‐2	49	20	(40.8)	29	(59.2)	
3–4	19	8	(42.1)	11	(57.9)	
cN						0.275
Negative	24	12	(50.0)	12	(50.0)	
Positive	44	16	(36.4)	28	(63.6)	
NG						0.851
1–2	21	9	(42.9)	12	(57.1)	
3	47	19	(40.4)	28	(59.6)	
Ki67						0.190
< 30%	10	6	(60.0)	4	(40.0)	
≥ 30%	58	22	(37.9)	36	(62.1)	
Histology						***0.049***
IDC	54	19	(35.2)	35	(64.8)	
Others	14	9	(64.3)	5	(35.7)	
Regimen						0.555
A → T	46	21	(45.7)	25	(54.3)	
Only A	19	6	(31.6)	13	(68.4)	
Only T	3	1	(33.3)	2	(66.7)	
LVI						0.243
Negative	38	18	(47.4)	20	(52.6)	
Positive	30	10	(33.3)	20	(66.7)	
Clinical therapeutic effects						0.243
PD or SD	30	10	(33.3)	20	(66.7)	
PR or CR	38	18	(47.4)	20	(52.6)	
Histological therapeutic effects						0.163
Grade 0–1a	36	12	(33.3)	24	(66.7)	
Grade 1b–2a	32	16	(50.0)	16	(50.0)	
Expression of each marker						
cGAS						0.705
Low	37	16	(43.2)	21	(56.8)	
High	31	12	(38.7)	19	(61.3)	
CD8						0.216
< 100	28	14	(50.0)	14	(50.0)	
≥ 100	40	14	(35.0)	26	(65.0)	
PD‐L1 (TC)						0.861
< 1%	38	16	(42.1)	22	(57.9)	
≥ 1%	30	12	(40.0)	18	(60.0)	
PD‐L1 (IC)						0.494
< 1%	12	6	(50.0)	6	(50.0)	
≥ 1%	56	22	(39.3)	34	(60.7)	
TIL						**0.057**
< 30%	56	26	(46.4)	30	(53.6)	
≥ 30%	12	2	(16.7)	10	(83.3)	

*Note:* Histological therapeutic effect is divided into Grade 0, 1a, 1b, 2a, 2b, and 3, with each therapeutic change meaning invalid, mild, moderate, severe, very severe, or complete response.

Abbreviations: A, anthracycline; cN, clinical lymph node status; CR, complete response; cT, clinical T classification; IDC, invasive ductal carcinoma; LVI, lymphovascular invasion; LVI was evaluated on surgical specimens; NG, nuclear grade; PD, progressive disease; PR, partial response; SD, stable disease; T, taxane. Italics values indicate statistical significance. Bold letters indicate clinical significance.

### 3.3 Association Between STING and cGAS Baseline Expression and Therapeutic Effects

We examined the association between STING and cGAS baseline expression in TCs and the response to NAC.

STING baseline expression did not show any association with clinical and histological therapeutic effects (*p* = 0.243 and 0.163, respectively) (Table 1). cGAS baseline expression showed an association with clinical therapeutic effects (*p* = 0.034) but not with histological therapeutic effects (*p* = 0.438) (Table S2).

### 3.4 STING And cGAS Expression Change by NAC

STING and cGAS expression were examined in surgical specimens to investigate changes in STING and cGAS expression by chemotherapy. Twenty three of 68 cases (33.8%) showed high STING expression persistently before and after chemotherapy. The expression was altered by NAC in 26 of 68 patients (38.2%), either as “low‐to‐high” (*n* = 9) or “high‐to‐low” (*n* = 17) (Table 2).

**TABLE 2:** Association between STING expression changes and clinicopathological factors.

Characteristics	Total	Low→low (*n* = 19)	Low→high (*n* = 9)	High→low (*n* = 17)	High→high (*n* = 23)	*p*
Pre‐treatment						
Menopause						0.561
Pre	31	10 (32.3)	3 (9.7)	6 (19.4)	12 (38.7)	
Post	37	9 (24.3)	6 (16.2)	11 (29.7)	11 (29.7)	
Tumor size						0.642
T1c	4	1 (25.0)	0	1 (25.0)	2 (50.0)	
T2	45	11 (24.4)	8 (17.8)	10 (22.2)	16 (35.6)	
T3	6	1 (16.7)	0	2 (33.3)	3 (50.0)	
T4	13	6 (46.2)	1 (7.7)	4 (30.8)	2 (15.4)	
Lymph nodes						0.632
Negative	24	9 (37.5)	3 (12.5)	5 (20.8)	7 (29.2)	
Positive	44	10 (22.7)	6 (13.6)	12 27.3	16 (36.4)	
Histology						***0.032***
IDC	54	15 (27.8)	4 (7.4)	14 (25.9)	21 (38.9)	
Others	14	4 (28.6)	5 (35.7)	3 (21.4)	2 (14.3)	
Regimen						0.941
A → T	46	14 (30.4)	7 (15.2)	11 (23.9)	14 (30.4)	
Only A	19	4 (21.1)	2 (10.5)	5 (26.3)	8 (42.1)	
Only T	3	1 (33.3)	0	1 (33.3)	1 (33.3)	
Post‐treatment						
CD8						***0.036***
< 100	17	9 (52.9)	3 (17.6)	1 (5.9)	4 (23.5)	
≥ 100	51	10 (19.6)	6 (11.8)	16 (31.4)	19 (37.3)	
PD‐L1 (IC)						**0.081**
< 1%		8 (47.1)	3 (17.6)	1 (5.9)	5 (29.4)	
≥ 1%		11 (21.6)	6 (11.8)	16 (31.4)	18 (35.3)	
LVI						**0.066**
Negative	38	14 (36.8)	4 (10.5)	10 (26.3)	10 (26.3)	
Positive	30	5 (16.7)	5 (16.7)	7 (23.3)	13 (43.3)	
Lymph nodes						**0.059**
Negative	41	16 (39.0)	6 (14.6)	8 (19.5)	11 (26.8)	
Positive	27	3 (11.1)	3 (11.1)	9 (33.3)	12 (44.4)	
Clinical therapeutic effects						0.485
PD or SD	30	8 (26.7)	2 (6.7)	8 (26.7)	12 (40.0)	
PR or CR	38	11 (28.9)	7 (18.4)	9 (23.7)	11 (28.9)	
Histological therapeutic effects						0.486
Grade 0–1a	36	9 (25.0)	3 (8.3)	10 (27.8)	14 (38.8)	
Grade 1b–2a	32	10 (31.3)	6 (18.8)	7 (21.9)	9 (28.1)	

*Note:* Histological therapeutic effect is divided into Grade 0, 1a, 1b, 2a, 2b, and 3, with each therapeutic change meaning invalid, mild, moderate, severe, very severe, or complete response.

Abbreviations: A, anthracycline; CR, complete response; IDC, invasive ductal carcinoma; LVI, lymphovascular invasion; PD, progressive disease; PR, partial response; SD, stable disease; T, taxane. Italics values indicate statistical significance. Bold letters indicate clinical significance.

In the adjacent tissues surrounding the cancer cells, STING and cGAS expression was almost universally high before and after chemotherapy.

The association between changes in STING and cGAS expression and clinicopathological factors was examined. The STING low to low group showed a lower number of CD8 positive cells in surgical specimens than the other groups (*p* = 0.036). The STING high to high group showed a trend toward more positive lymphovascular invasion (LVI) (*p* = 0.066; Table 2). No associations of changes in STING expression were observed with other factors including chemotherapy regimens or therapeutic effects. Changes in cGAS expression showed no association with clinicopathological factors other than the histological type of cancer (Table S3).

### 3.5 Association of STING and cGAS Expression With Survival

**FIGURE 3:**
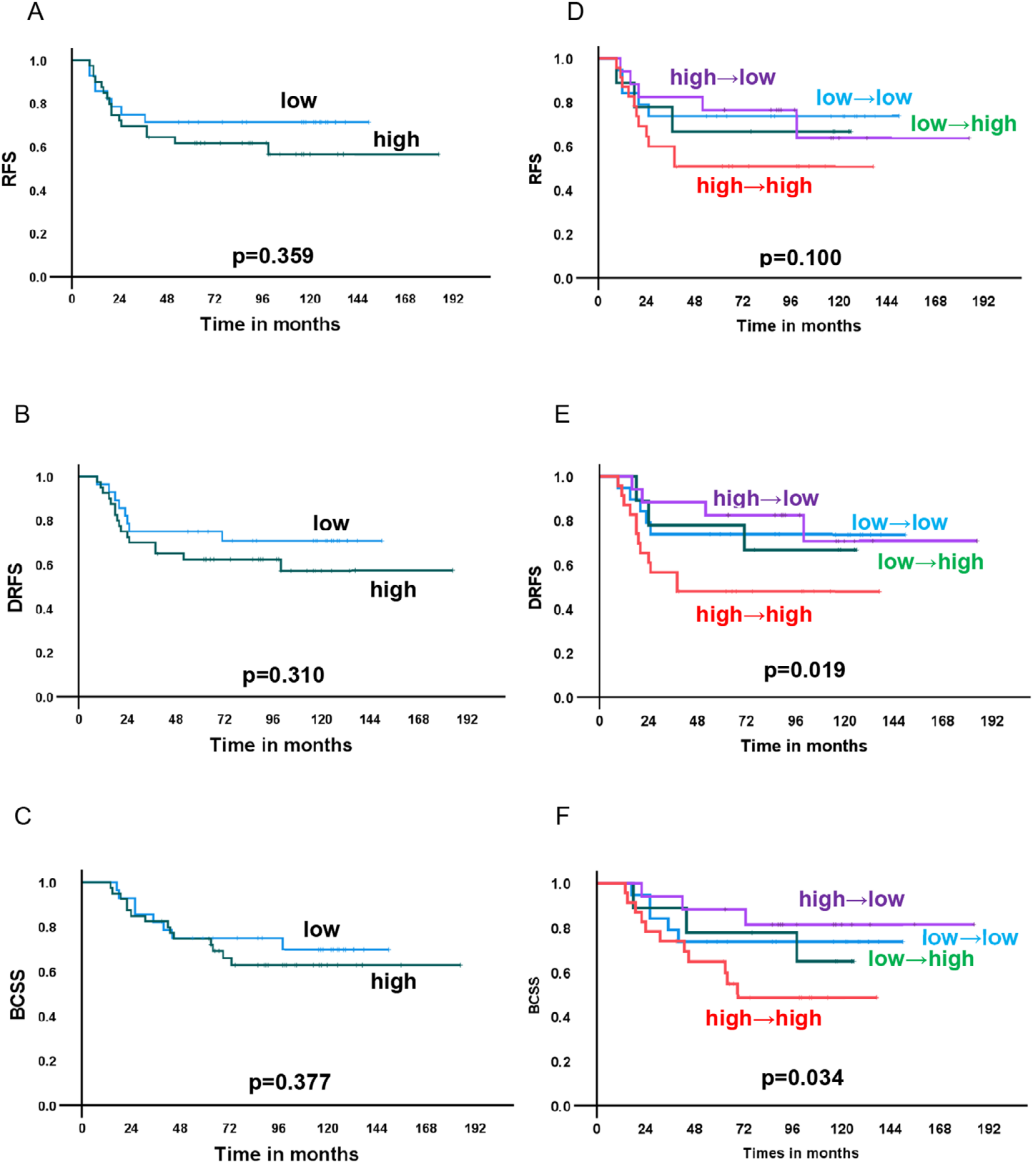
Survival curves by STING expression in biopsy specimens and by STING expression change by neoadjuvant chemotherapy. Recurrence‐free survival (RFS), distant recurrence‐free survival (DRFS), and breast cancer‐specific survival (BCSS) were drawn according to STING expression and its changes by neoadjuvant chemotherapy. (A–C): RFS (A), DRFS (B), and BCSS (C) by high and low STING expression in the biopsy specimen. (D–F): RFS (D), DRFS (E), and BCSS (F) by changes in STING expression before and after neoadjuvant chemotherapy.

**FIGURE 4:**
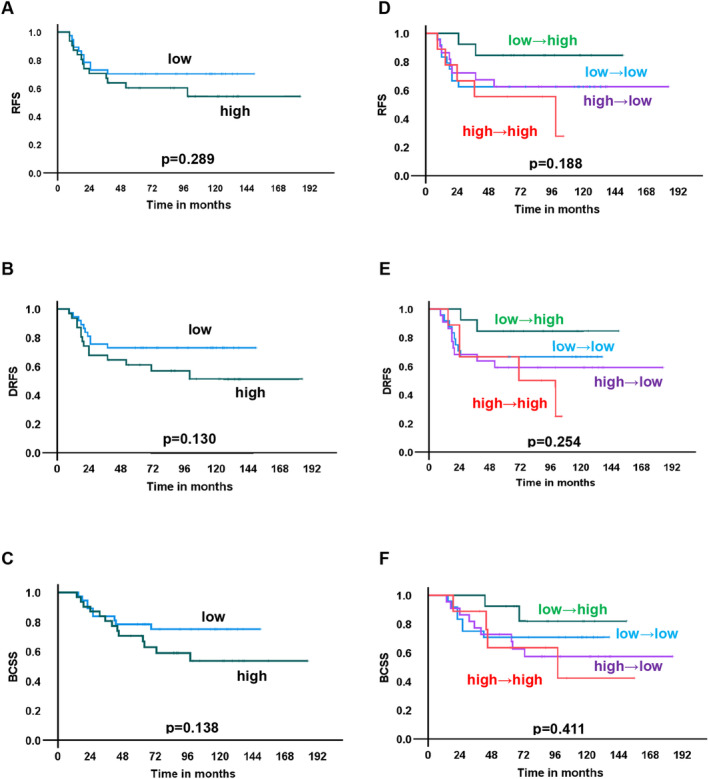
Survival curves by cGAS expression in biopsy specimens and by cGAS expression change by neoadjuvant chemotherapy. Recurrence‐free survival (RFS), distant recurrence‐free survival (DRFS), and breast cancer‐specific survival (BCSS) were drawn according to cGAS expression and its changes by neoadjuvant chemotherapy. (A–C): RFS (A), DRFS (B), and BCSS (C) by high and low cGAS expression in the biopsy specimen. (D–F): RFS (D), DRFS (E), and BCSS (F) by changes in cGAS expression before and after neoadjuvant chemotherapy.

The median follow‐up period was 115 months (range: 14–185 months). We examined survival according to STING expression at baseline. Patients with high STING expression at baseline tended to have a poorer prognosis of recurrence‐free survival (RFS), distant recurrence‐free survival (DRFS), and breast cancer‐specific survival (BCSS), although the differences in survival between those with high and low STING expression were not significant (Figure 3A–C). A similar trend was observed for cGAS (Figure 4A–C).

Next, the association between changes in STING and cGAS expression and survival was examined. Patients with “high‐to‐high” STING expression showed a significantly worse prognosis than that of the others in terms of DRFS and BCSS (*p* = 0.019, and 0.034, respectively) and a similar trend without statistical significance for RFS (*p* = 0.100) (Figure 3D–F). Because patients with “high‐to‐high” STING expression had poor survival, the therapeutic effects of NAC were compared between patients with “high‐to‐high” STING expression and the others. There was no difference in clinical and histological therapeutic effect between the “high‐to‐high” group and the others (Table S4). No association between changes in cGAS expression and prognosis was observed in terms of RFS, DRFS and BCSS (Figure 4D–F).

### 3.6 Univariate and Multivariate Cox Regression Analyses for Changes in STING Expression

To investigate the prognostic impact of changes in STING expression, we conducted a univariate Cox regression analysis (Table 3). Having “high‐to‐high” STING expression was negatively correlated with DRFS (*p* = 0.024) and BCSS (*p* = 0.040). Some other factors, including LVI and lymph node status in surgical specimens, were also associated with survival. Multivariate Cox regression analysis revealed that a maintained high STING expression (high‐to‐high) was a poor prognostic factor for DRFS and BCSS independent of other factors, including LVI, lymph node status, and clinical therapeutic effects (*p* = 0.024 and 0.014, respectively).

**TABLE 3:** Univariate and Multivariate Cox regression analysis.

Variables	Univariate analysis									Multivariate analysis								
	RFS			DRFS			BCSS			RFS			DRFS			BCSS		
	HR	95% CI	*p*	HR	95% CI	*p*	HR	95% CI	*p*	HR	95% CI	*p*	HR	95% CI	*p*	HR	95% CI	*p*
STING changes																		
High→high vs. other	1.929	0.862–4.317	0.110	2.526	1.130–5.645	***0.024***	2.411	1.042–5.578	***0.040***	1.600	0.672–3.814	0.289	2.744	1.141–6.597	***0.024***	3.272	1.269–8.434	***0.014***
CD8																		
< 100 vs. 100 ≤	2.125	0.929–4.863	*0.074*	2.153	0.941–4.929	*0.069*	2.584	1.102–6.062	***0.029***	4.133	1.476–11.574	***0.007***	4.992	1.783–13.979	***0.002***	7.756	2.543–23.655	***< 0.001***
LVI																		
Positive vs. negative	3.449	1.471–8.088	***0.004***	3.214	1.372–7.531	***0.007***	2.470	1.035–5.893	***0.042***	2.287	0.938–5.577	*0.069*	1.971	0.806–4.823	0.137	1.474	0.600–3.617	0.397
Menopause																		
Pre vs. post	1.068	0.478–2.386	0.873	1.026	0.459–2.292	0.951	1.238	0.536–2.858	0.617	ー	ー	ー	ー	ー	ー	ー	ー	ー
Clinical Tumor size																		
T3‐4 vs. T1c‐2	2.061	0.915–4.640	0.081	2.060	0.915–4.639	0.081	1.963	0.839–4.596	0.120	ー	ー	ー	ー	ー	ー	ー	ー	ー
Lymph node status																		
Positive vs. negative	3.329	1.449–7.649	***0.005***	3.236	1.411–7.423	***0.006***	3.440	1.437–8.234	***0.006***	4.272	1.561–11.696	***0.005***	4.186	1.548–11.318	***0.005***	6.075	2.122–17.395	***< 0.001***
Clinical therapeutic effect																		
PD or SD vs. PR or CR	3.018	1.290–7.059	***0.011***	2.439	1.067–5.576	***0.035***	1.970	0.841–4.615	*1.600*	1.600	0.672–3.814	0.289	1.651	0.704–3.871	0.249	1.472	0.616–3.522	0.385

Abbreviations: BCSS, breast cancer‐specific survival; CR, complete response; DRFS, distant recurrence‐free survival; LVI, lymphovascular invasion; PD, progressive disease; PR, partial response; RFS, recurrence‐free survival; SD, stable disease. Italics values indicate statistical significance. Bold letters indicate clinical significance.

## 4 Discussion

This study examined STING protein expression in human breast cancer tissues before and after NAC and found that “high‐to‐high” STING expression was a poor prognostic factor for DRFS and BCSS, independent of LVI, CD8 and lymph node status in TNBCs. In addition, high STING expression in TCs at baseline showed a trend toward more TILs, which is consistent with the biological function of STING [14].

Previous studies have reported conflicting roles of the STING pathway in cancer progression. cGAMP binds to STING in the endoplasmic reticulum and eventually reaches the Golgi apparatus, where it recruits TANK‐binding kinase 1 (TBK1). This interaction triggers TBK1‐mediated phosphorylation of STING, leading to the recruitment and activation of interferon regulatory factor 3 (IRF3) and NF‐κB. This activation induces the production of type I IFN, increases the expression of inflammatory cytokines, activates CD8^+^ T cells and NK cells, and ultimately promotes endogenous antitumor effects [14, 39, 40].

However other reports show that chronic stimulation of the STING pathway can induce chronic inflammation, promote tumor growth, and may induce an immunosuppressive tumor microenvironment [39, 40, 41, 42, 43]. In this study, the expression of cGAS and STING did not correlate with the pathological response to treatment, but rather “high‐to‐high” STING expression indicated a poor survival, supporting the tumor promoting effects of the STING pathway [39, 40, 41, 42, 43, 44] rather than the anti‐tumor effects [14, 39, 40] and suggesting that tumors with “high‐to‐high” STING expression harbor a chronic inflammatory state, which may promote tumor progression. This notion seems supported by the finding of a trend toward higher LVI in patients with “high‐to‐high” STING expression because chronic inflammation causes lymphangiogenesis [44]. Chronic inflammation reportedly induces not only cancer growth but also invasion and metastasis [44], which may explain, to some extent, a poor prognosis in patients with “high‐to‐high” STING expression.

PD‐L1 induction and the antitumor immune response induced by STING are among the rationales for clinical trials of treatment using the STING agonist alone or in combination with immune checkpoint inhibitors (ICI) for locally advanced or metastatic solid tumors that are currently underway [45]. Because patients in this study did not receive an ICI for NAC, current NAC regimens containing an ICI for high‐risk TNBC may improve the prognosis of patients with “high‐to‐high” STING expression or reduce the number of those patients, which should be further examined in a future study.

Recently an IHC‐based classification of TNBC has been proposed [46]. TNBCs are classified into five subtypes according to the IHC results: IHC‐based luminal androgen receptor (AR+), IHC‐based immunomodulatory (AR−, CD8^+^), IHC‐based basal‐like immune‐suppressed (AR−, CD8−, FOXC1+), IHC‐based mesenchymal (AR−, CD8−, FOXC1−, DCLK1+), and IHC‐based unclassifiable (AR−, CD8−, FOXC1−, DCLK1−). This classification is not only prognostic but also indicative of therapeutic strategies. In order to further understand the biology of TNBC, it would be of clinical value to examine STING expression based on this classification in the future study.

This study has several limitations. First, only the protein expression of STING and cGAS was examined. Thus, their activation was not assessed in clinical samples and not showing relationship to their molecular features. Studies with antibodies to detect phosphorylated STING or to examine the factors of the STING downstream signaling pathway in more detail will further clarify the clinical relevance of STING activity. Secondly, several different chemotherapy regimens were used. However, the changes in STING expression did not differ depending on the regimen (Table 2), which may suggest the minimal impact of different regimens on the results. Regimens containing an ICI were not used in this study and need to be examined in the future study. Thirdly, this study is a single‐center retrospective analysis, which might have caused selection bias and information bias, and resulted in a small sample size. Thus, cautious interpretation of the results is required. A larger study is needed to validate and generalize our results. Fourthly, patients with pCR and RCB1 were excluded because protein expression in post‐treatment cancer cells could not be examined. Patients with pCR or RCB1 have a favorable prognosis while those without pCR nor RCB1 show a relatively poor prognosis, among whom we further tried to identify those at a high risk of recurrence, who would require additional treatment strategies. Fifth, the assessment for PD‐L1 was performed with the antibody, SP142, which might have resulted in a lower sensitivity for PD‐L1 expression in TCs. SP142 was selected because it was the only antibody clinically available at the time of the start of this study. It is clinically important to perform future studies using other antibodies including 22C3 to examine the association between PD‐L1 expression in TC and STING or cGAS expression in breast cancer tissues.

## 5 Conclusion

Patients with sustained high expression of STING before and after NAC had a significantly poorer prognosis than those with other changes in STING expression and will be a target population for new treatment strategies once the results are validated in a larger study through multi‐center collaborations including patients treated with ICI‐containing regimens.

## Author Contributions

**Tetsuyo Maeda:** data curation (equal), formal analysis (equal), investigation (equal), methodology (equal), project administration (equal), software (equal), validation (equal), visualization (equal), writing – original draft (lead). **Makiko Ono:** conceptualization (equal), investigation (equal), methodology (equal), software (equal), supervision (equal), writing – original draft (equal), writing – review and editing (equal). **Tomo Osako:** investigation (equal), methodology (equal), writing – review and editing (equal). **Tomohiro Chiba:** formal analysis (equal), investigation (equal), methodology (equal), supervision (equal), writing – review and editing (equal). **Satoko Baba:** investigation (equal), methodology (equal), writing – review and editing (equal). **Asumi Iesato:** investigation (equal), methodology (equal), writing – review and editing (equal). **Yukinori Ozaki:** investigation (equal), methodology (equal), writing – review and editing (equal). **Yuka Inoue:** methodology (equal), supervision (equal), writing – review and editing (equal). **Natsue Uehiro:** investigation (equal), methodology (equal), writing – review and editing (equal). **Yoko Takahashi:** investigation (equal), methodology (equal), writing – review and editing (equal). **Takayuki Kobayashi:** investigation (equal), methodology (equal), writing – review and editing (equal). **Takahiro Kogawa:** investigation (equal), methodology (equal), writing – review and editing (equal). **Tomohiko Ohta:** conceptualization (equal), methodology (equal), supervision (equal), writing – review and editing (equal). **Shigehisa Kitano:** conceptualization (equal), investigation (equal), methodology (equal), supervision (equal), writing – review and editing (equal). **Takayuki Ueno:** conceptualization (equal), methodology (equal), software (equal), supervision (equal), writing – original draft (equal). **Shinji Ohno:** conceptualization (equal), supervision (equal), writing – review and editing (equal).

## Ethics Statement

Ethical approval to report this study was obtained from the Institutional Review Board of Cancer Institute Hospital (registration number: 2018‐GA‐1220) and was conducted according to the principles of the Declaration of Helsinki.

## Consent

This study does not require patient consent because of its retrospective design according to the Institutional Review Board.

## Conflicts of Interest

The authors declare no conflicts of interest.

## Supporting information


Data S1.

## Data Availability

The data that support the findings of this study are available from the corresponding author upon reasonable request.
