# Supplementary material for: Prognostic Impact of Stimulator of Interferon Genes Expression in Triple Negative Breast Cancer
Source: Cancer Med. 2025 Feb 18;14(4):e70666. doi: 10.1002/cam4.70666 (PMC11836529; doi:10.1002/cam4.70666)
Supplement: Supplementary file 1 — Data S1. [file CAM4-14-e70666-s001.docx]

**Supplementary Table 1. Immunohistochemistry protocols**

| **Antigen** | **Clone and origin** | **Dilution, retrieval** | **Source, reference** | **Assessment** | **Cut-off** |
| --- | --- | --- | --- | --- | --- |
| ER | SP-1, Rabbit mAb | RTU, pH9,64min | Roche | nuclear | Negative < 1%; and positive ≧ 1% |
| PgR | 1E2, Rabbit mAb | RTU, pH9, 64min | Roche | nuclear | Negative < 1%; and positive ≧ 1% |
| HER2 | 4B5, Rabbit mAb | RTU, pH9, 64min | Roche | cellular membrane | positive; 3+ or (2+ and DISH positive) |
| Ki67 | MIB-1, Mouse mAb | 1:200, pH9, 20min | DAKO | nuclear | Low < 10%, 10 ≦ intermediate ≦ 30%, 30% < high |
| CD8 | C8/144B, Mouse mAb | 1:3, pH9, 20min | Nichirei Biosciences, 413201 | cellular membrane | Positive ≧ 100 positive cells / field, in 5 hot spots at 400x focus |
| PD-L1 | SP142, Rabbit mAb | 1:400, pH9, 40min | Abcam, 228462 | cellular membrane | TC, IC≧1% |
| cGAS | MB21 D1, Rabbit-pAb | 1:1000, pH9.0, 30min | Sigma, HPA031700 | cytoplasm | H-score low ≦ 200, high > 200 |
| STING | TMEM173, Rabbit-pAb | 1:1500, pH9.0, 30min | Proteintech, 19851-1-AP | cytoplasm | H-score low ≦ 70, high > 70 |

ER: estrogen receptor, PgR: progesterone receptor, HER2: human epidermal receptor 2, PD-L1: programmed cell death ligand 1,

TC: tumor cell, IC: immune cell, cGAS: cyclic GMP-AMP synthase, STING: the stimulator of interferon genes

**Supplementary Table 2. Association between cGAS expression in biopsy specimens and clinicopathological characteristics.**

| **Characteristics** | **Total (n = 68)** | **cGAS low (n = 37)** | | **cGAS high (n = 31)** | | p |
| --- | --- | --- | --- | --- | --- | --- |
| **Menopause** |  |  |  |  |  | 0.948 |
| pre- | 31 | 17 | (54.8) | 14 | (45.2) |  |
| post- | 37 | 20 | (54.1) | 17 | (45.9) |  |
| **cT** |  |  |  |  |  | ***0.019*** |
| 1c-2 | 49 | 31 | (63.3) | 18 | (36.7) |  |
| 3-4 | 19 | 6 | (31.6) | 13 | (68.4) |  |
| **cN** |  |  |  |  |  | 0.323 |
| negative | 24 | 15 | (62.5) | 9 | (37.5) |  |
| positive | 44 | 22 | (50.0) | 22 | (50.0) |  |
| **NG** |  |  |  |  |  | 0.071 |
| 1-2 | 21 | 8 | (38.1) | 13 | (61.9) |  |
| 3 | 47 | 29 | (61.7) | 18 | (38.4) |  |
| **Ki67** |  |  |  |  |  | 0.701 |
| <30% | 10 | 6 | (60.0) | 4 | (40.0) |  |
| ≧30% | 58 | 31 | (53.4) | 27 | (46.6) |  |
| **Histology** |  |  |  |  |  | 0.330 |
| IDC | 54 | 31 | (57.4) | 23 | (42.6) |  |
| others | 8 | 6 | (42.9) | 8 | (57.1) |  |
| **Regimen** |  |  |  |  |  | 0.075 |
| A→T | 46 | 24 | (52.2) | 22 | (47.8) |  |
| only A | 19 | 13 | (68.4) | 6 | (31.6) |  |
| only T | 3 | 0 |  | 3 | (100.0) |  |
| **LVI** |  |  |  |  |  | 0.255 |
| negative | 38 | 23 | (60.5) | 15 | (39.5) |  |
| positive | 30 | 14 | (46.7) | 16 | (53.3) |  |
| **Clinical therapeutic effects** | |  |  |  |  | ***0.034*** |
| PD or SD | 30 | 12 | (40.0) | 18 | (60.0) |  |
| PR or CR | 38 | 25 | (65.8) | 13 | (34.2) |  |
| **Histological therapeutic effects** | |  |  |  |  | 0.438 |
| Grade0-1a | 36 | 18 | (50.0) | 18 | (50.0) |  |
| Grade1b-2a | 32 | 19 | (59.4) | 13 | (40.6) |  |
|  |  |  |  |  |  |  |
| **Expression of each marker** |  |  |  |  |  |  |
| **CD8** |  |  |  |  |  | 0.383 |
| <100 | 28 | 17 | (60.7) | 11 | (39.3) |  |
| ≧100 | 40 | 20 | (50.0) | 20 | (50.0) |  |
| **PD-L1(TC)** |  |  |  |  |  | 0.740 |
| TC<1% | 38 | 20 | (52.6) | 18 | (47.4) |  |
| TC≧1% | 30 | 17 | (56.7) | 13 | (43.3) |  |
| **PD-L1(IC)** |  |  |  |  |  | 0.735 |
| IC<1% | 12 | 6 | (50.0) | 5 | (50.0) |  |
| IC≧1% | 56 | 31 | (55.4) | 25 | (44.6) |  |
| **TIL** |  |  |  |  |  | 0.348 |
| <30% | 56 | 29 | (51.8) | 27 | (48.2) |  |
| ≧30% | 12 | 8 | (66.7) | 4 | (33.3) |  |

cT: clinical T classification, cN: clinical lymph node status, NG: nuclear grade, IDC: invasive ductal carcinoma, A: anthracycline, T: taxane, LVI: lymphovascular invasion, LVI was evaluated on surgical specimens. PD: progressive disease, SD: stable disease, PR: partial response, CR: complete response Histological therapeutic effect is divided into Grade 0, 1a, 1b, 2a, 2b, and 3, with each therapeutic change meaning invalid, mild, moderate, severe, very severe, or complete response.

**Supplementary Table 3. Association between cGAS expression changes and clinicopathological factors**

|  |  |  |  |  |  |  |  |  |  |  |
| --- | --- | --- | --- | --- | --- | --- | --- | --- | --- | --- |
| **Characteristics** | **Total** | **low→low (n = 24)** | | **low→high (n = 13)** | | **high→low (n = 22)** | | **high→high (n = 9)** | | **p** |
| **Pre-treatment** |  |  |  |  |  |  |  |  |  |  |
| **Menopause** |  |  |  |  |  |  |  |  |  | 0.461 |
| pre- | 31 | 13 | (41.9) | 4 | (12.9) | 11 | (35.5) | 3 | (9.7) |  |
| post- | 37 | 11 | (29.7) | 9 | (24.3) | 11 | (29.7) | 6 | (16.2) |  |
| **Tumor size** |  |  |  |  |  |  |  |  |  | 0.339 |
| T1c | 4 | 1 | (25.0) | 1 | (25.0) | 1 | (25.0) | 1 | (25.0) |  |
| T2 | 45 | 21 | (46.7) | 8 | (17.8) | 11 | (24.4) | 5 | (11.1) |  |
| T3 | 6 | 0 |  | 1 | (16.7) | 4 | (66.7) | 1 | (16.7) |  |
| T4 | 13 | 2 | (15.4) | 3 | (23.1) | 6 | (46.2) | 2 | (15.4) |  |
| **Lymph nodes** |  |  |  |  |  |  |  |  |  | 0.424 |
| negative | 24 | 10 | (41.7) | 5 | (20.8) | 8 | (33.3) | 1 | (4.2) |  |
| positive | 44 | 14 | (31.8) | 8 | (18.2) | 14 | (31.8) | 8 | (18.2) |  |
| **Histology** |  |  |  |  |  |  |  |  |  | 0.039 |
| IDC | 54 | 21 | (38.9) | 10 | (18.5) | 19 | (35.2) | 4 | (7.4) |  |
| others | 14 | 3 | (21.4) | 3 | (21.4) | 3 | (21.4) | 5 | (35.7) |  |
| **Regimen** |  |  |  |  |  |  |  |  |  | 0.175 |
| A→T | 46 | 15 | (32.6) | 9 | (19.6) | 14 | (30.4) | 8 | (17.4) |  |
| only A | 19 | 9 | (47.4) | 4 | (21.1) | 5 | (26.3) | 1 | (5.3) |  |
| only T | 3 | 0 |  | 0 |  | 3 | (100.0) | 0 |  |  |
| **Post-treatment** |  |  |  |  |  |  |  |  |  |  |
| **CD8** |  |  |  |  |  |  |  |  |  | 0.588 |
| <100 | 17 | 5 | (29.4) | 4 | (23.5) | 7 | (41.2) | 1 | (5.9) |  |
| ≧100 | 51 | 19 | (37.3) | 9 | (17.6) | 15 | (29.4) | 8 | (15.7) |  |
| **PD-L1 (IC)** |  |  |  |  |  |  |  |  |  | 0.250 |
| <1% |  | 3 | (17.6) | 4 | (23.5) | 6 | (35.3) | 4 | (23.5) |  |
| ≧1% |  | 21 | (41.2) | 9 | (17.6) | 16 | (31.4) | 5 | (9.8) |  |
| **LVI** |  |  |  |  |  |  |  |  |  | 0.585 |
| negative | 38 | 16 | (42.1) | 7 | (18.4) | 11 | (28.9) | 4 | (10.5) |  |
| positive | 30 | 8 | (26.7) | 6 | (20.0) | 11 | (36.7) | 5 | (16.7) |  |
| **Lymph nodes** |  |  |  |  |  |  |  |  |  | 0.162 |
| negative | 41 | 18 | (43.9) | 7 | (17.1) | 13 | (31.7) | 3 | (7.3) |  |
| positive | 27 | 6 | (22.2) | 6 | (22.2) | 9 | (33.3) | 6 | (22.2) |  |
| **Clinical therapeutic effects** | |  |  |  |  |  |  |  |  | 0.208 |
| PD or SD | 30 | 8 | (26.7) | 4 | (13.3) | 13 | (43.3) | 5 | (16.7) |  |
| PR or CR | 38 | 16 | (42.1) | 9 | (23.7) | 9 | (23.7) | 4 | (10.5) |  |
| **Histological therapeutic effects** | |  |  |  |  |  |  |  |  | 0.497 |
| Grade0-1a | 36 | 13 | (36.1) | 5 | (13.9) | 14 | (38.9) | 4 | (11.1) |  |
| Grade1b-2a | 32 | 11 | (34.4) | 8 | (25.0) | 8 | (25.0) | 5 | (15.6) |  |

IDC: invasive ductal carcinoma, A: anthracycline, T: taxane, LVI: lymphovascular invasion, PD: progressive disease, SD: stable disease, PR: partial response, CR: complete response, Histological therapeutic effect is divided into Grade 0, 1a, 1b, 2a, 2b, and 3, with each therapeutic change meaning invalid, mild, moderate, severe, very severe, or complete response.

**Supplementary Table 4. Association between STING expression changes and the therapeutic effects**

|  |  |  |  |  |  |  |
| --- | --- | --- | --- | --- | --- | --- |
|  | **Total (n = 68)** | **high→high (n = 23)** | | **Other (n = 45)** | | **p** |
|  | **No.** | **No.** | **%** | **No.** | **%** |  |
| **Clinical therapeutic effects** |  |  |  |  |  | 0.339 |
| PD or SD | 30 | 12 | (60.0) | 18 | (40.0) |  |
| PR or CR | 38 | 11 | (28.9) | 27 | (71.1) |  |
| **Histological therapeutic effects** |  |  |  |  |  | 0.349 |
| Grade0-1a | 36 | 14 | (38.9) | 22 | (61.1) |  |
| Grade1b-2a | 32 | 9 | (28.1) | 23 | (71.9) |  |

PD: progressive disease, SD: stable disease, PR: partial response, CR: complete response
